# Normal Values of Metatarsal Parabola Arch in Male and Female Feet

**DOI:** 10.1155/2014/505736

**Published:** 2014-02-06

**Authors:** Gabriel Domínguez-Maldonado, Pedro V. Munuera-Martinez, José Manuel Castillo-López, Javier Ramos-Ortega, Manuel Albornoz-Cabello

**Affiliations:** ^1^Department of Podiatrics, University of Seville, Seville, Spain; ^2^Teaching Center of Physiotherapy and Podiatry. Calle Avicena s/n, 41009 Seville, Spain

## Abstract

There is not any method to measure metatarsal protrusion in the whole metatarsal. The aim of this research is to know the normal metatarsal parabola in male and female feet. The system of measurement devised by Hardy and Clapham to evaluate the protrusion between metatarsals I and II was adapted to study the whole metatarsal parabola and applied to the five metatarsals of 169 normal feet, 72 female feet and 97 male feet. Authors measured all metatarsal protrusion relative to metatarsal II. The results obtained show a female metatarsal protrusion relative to metatarsal II of +1.27% for metatarsal I, −3.36% for metatarsal III, −8.34% for metatarsal IV, and −15.54% for metatarsal V. Data obtained for male metatarsal parabola were +0.5% for metatarsal I, −3.77 for metatarsal III, −9.57 for metatarsal IV, and −17.05 for metatarsal V. Differences between both metatarsal parabola were significant.

## 1. Introduction

The metatarsal parabola has been the object of study by various authors [1]. Most of the studies published on the values of metatarsal protrusion refer exclusively to the relationship between metatarsals I and II, using the distance (expressed in mm) between the tangents to the two metatarsal heads as protrusion value. These works include those of Morton [2], Harris and Beath [3, 4], Hardy and Clapham [5], and LaPorta et al. [6], in which the different authors cite various systems for measuring metatarsal I-II protrusion and establish various values of normality. Valley and Reese [7] designed three different systems of measurement to evaluate the protrusion of the lesser metatarsals, although none of them independently achieved complete analysis of the metatarsal parabola.

On the other hand, anthropometric differences cited by various authors regarding the alignment of the lower extremity suggest the need to compare the mean values of the metatarsal arch in men and women. Testud and Latarjet [8] established 16 differences between the pelvis in men and women, such as differences in the cervicodiaphyseal angle of the femur. There are also references concerning the difference of angulation of femoral anteversion [9–11] and of physiological genu valgo depending on gender [12–14]. With regard to the foot, significant differences were found by Steele [15] in the size of astragalus and calcaneus, by Smith [16] in the size of metatarsals and toes, and by Ferrari et al. [17] in the orientation of the first ray in the transverse plane.

Gender-dependent differences have been described regarding the functionality of the lower extremity. Staheli et al. [18] found significant differences in the internal rotation of the hip, the internal rotator pattern [19], and the angle of gait [20–25], and even in plantar pressures during gait [26, 27].

The aims of the present study were (1) to obtain the mean values of metatarsal protrusion—with respect to metatarsal II—for all the metatarsals, using the method described by Hardy and Clapham, (2) to compare the mean values of metatarsal protrusion obtained with that system between men and women, and (3) to evaluate the reliability of the radiological measurements, made using computer programs. These values are important to understand the biomechanics of the forefoot and to know the normal shape and function of the foot on orthopedics and surgery treatments.

## 2. Materials and Methods

This one is an observational research, to measure metatarsal protrusion in male and female feet and state normal values.

Dorsoplantar radiographic plates, made under load with a focal inclination of 15° with respect to vertical, are used. The study consists of 169 feet (87 right feet and 82 left feet; 72 female feet and 97 male feet) of 105 volunteers (63 men and 42 women), podiatry students at Seville University. In several cases only one foot was used. All subjects provided written consent.

The criteria for inclusion were as follows: more than 20 years old, without deformities of the forefoot (HL, extension MTF I > 65°; HAV, claw toes, etc.), without degenerative osteoarticular disease or muscular imbalance, without signs of alterations in the forefoot load distribution, with absence of foot pain, without previous surgery of the foot, and without trauma to the foot in the previous 12 months.

The mean age of the subjects taking part in the study was 23.6 ± 2.7 years old, with no subject being under 20 years old (and the skeleton of the foot still developing).

The radiological plates were scanned using a radiological scanner and digitized, and radiological measurements were made with the program AUTOCAD 2006.

The metatarsal protrusion is measured using the method Hardy and Clapham [5] described to determine MTT I-II protrusion, applying it in our case to the rest of the metatarsals. This consists of determining the protrusion of MTT I, III, IV, and V relative to the length of MTT II. We use MTT II as reference for various reasons:

- it is considered the longitudinal axis of the metatarsus, commonly used as a reference for different radiological measurements of the forefoot [28].
- Due to the conformation of the second metatarsocuneiform joint, the fit of the metatarsal between the first and third cuneiforms, and the resulting limitation to the mobilization of this joint, it is the most stable metatarsal, with little capacity to become malaligned, affecting its protrusion.
- It is not affected by brachymetatarsia usually [29]. Brachymetatarsia is an abnormal shortness of the metatarsals that can affect any of the five metatarsal, but the one most frequently involved is the 4th. The deformity is not very common, and its incidence has been determined as between 1 in 1820 and 1 in 4586 of the population [29].

Firstly, we trace the transverse axis of the tarsus, with a line joining the most posterior point of the scaphoid tubercle with the posterior surface of the proximal articular facet of the cuboid.

**Figure 1:**
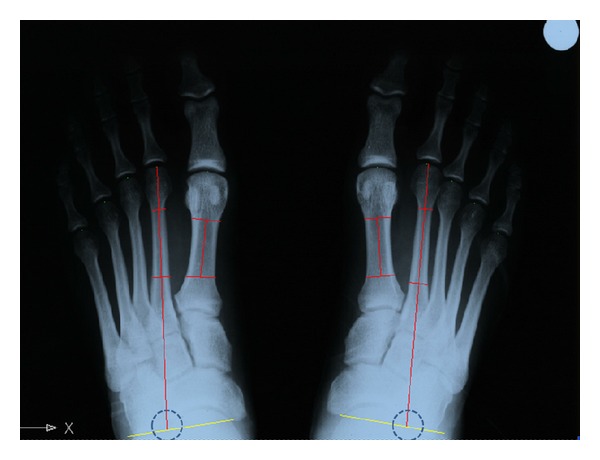
The intersection between the diaphyseal axis of MTT II and the transverse axis of the tarsus was considered as point of rotation to project the tangent to the metatarsal heads.

We use the intersection between the diaphyseal axis of MTT II and the transverse axis of the tarsus as point of rotation to project the tangent to the metatarsal heads on the axis of ray II (Figure 1).

**Figure 2:**
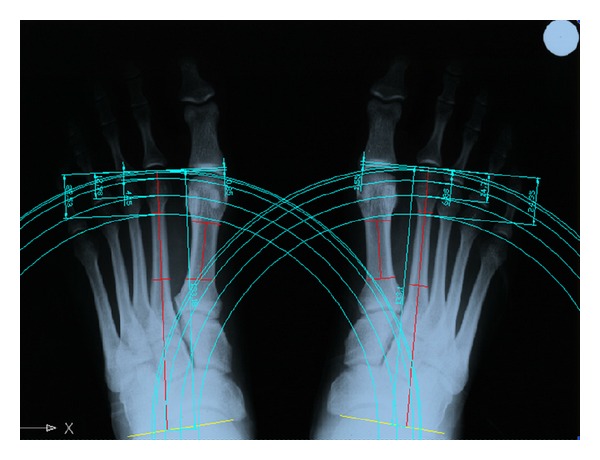
Values of metatarsal protrusion was measured as the distances between the arcs of circumference tangent to the most distal points of each metatarsal head and the most distal point of the head of MTT II.

The distances between the arcs of circumference tangent to the most distal points of the metatarsal heads are expressed as percentage of the distance between the most distal point of the head of MTT II and the line of the transverse axis of the tarsus (ray II length) (Figure 2).

The diaphyseal axes were traced as indicated by Coughlin et al. [30], using the midpoints of the proximal and distal metaphyseal zones, in an area between 0.5 and 1 cm from the articular surface in the case of the phalanges, and between 1 and 2 cm in that of the metatarsals.

We studied main values for all variables (metatarsal protrusion angle for I, III, IV, and V metatarsals) and the Student's *t*-test to compare male and female groups.

To test the reliability of the measurements, they were made three times in 8 feet selected at random, at intervals of one week between observations. In order to determine the intrareliability, we used these three observations to obtain intraclass coefficient of correlation for all variables.

All measurements were made by one evaluator. The statistical analysis of the data was performed using the program SPSS 12.0 for WINDOWS.

## 3. Results and Discussion

The statistical analysis established the mean value and the standard deviations for the protrusions of MTT I, III, IV, and V relative to MTT II. The results are displayed in Tables 1 and 2.

The values of the metatarsal protrusion angle showed significant differences (*P* < 0.05) depending on gender (Tables 3 and 4).

The results of the test of intraclass correlation are presented in Table 5.

With the system of measurement of metatarsal protrusion proposed and tested in this study, the authors have found mean values of +0.83% ± 2.19 and +0.94 ± 2.71 mm of metatarsal protrusion between MTT II and I. The pattern of metatarsal protrusion reflected in the study contradicts the data from Nilsonne's study in 1930 [31] of 497 feet included in a control group, which determined that 52.2% presented a shorter MTT I, against 34.4% showing Index Plus.

Viladot [32] also refers to the Index Minus metatarsal formula, with an MTT I anatomically shorter than MTT II in 56% of the population, and 16% in the Index Plus formula.

The mean values of absolute protrusion found by the authors are in agreement with the values of normality established as ±2 mm by Weissman in 1989 [33], Palladino in 1990 [34], and Heden and Sorto Jr. in 1994 [35]. The data obtained in the present study are also similar to those cited by Hardy and Clapham in 1951 [5], Munuera-Martínez et al. in 2004 [29], and Domínguez et al. in 2006 [36].

Munuera-Martínez et al. [29] established in a control group of 252 subjects a mean value for MTT I-II protrusion of 2 mm, increasing to 4 mm in the HAV-affected group studied. The latter found a mean value of MTT II–IV relative protrusion of –13.74 mm for men and –10.24 mm for women in the control group, against the –12.33 mm for men and −9.60 mm for women found in the present study. In 2006, the authors [36] found mean values of metatarsal protrusion in a specimen of 52 feet of +1.22% for metatarsal I, −3.84% for metatarsal III, −9.66% for metatarsal IV, and –16.91% for metatarsal V (there were no differences between male and female feet). These mean values are close to data from the current study showing mean values of metatarsal protrusion of +0.94% for metatarsal I, −4.44% for metatarsal III, −11.17% for metatarsal IV, and −20.21% for metatarsal V.

The authors have found higher values of metatarsal protrusion than those reported by Valley and Reese in 1991 [7]. Due to the difference between the system for measuring metatarsal protrusion proposed here and the systems described by Valley and Reese, the results obtained in the two studies cannot be considered comparable.

The anatomical differences between the male and female skeleton are not only due to size differences that can be presented by specific osseous parts—for instance the male cranium is larger, with more-marked osseous reliefs (glabella, ciliary arches, inion, occipital condyles, mastoid and styloid apophyses, etc.) [8]—the female pelvis is broader [8], and men present larger bones of the rearfoot [15] and metatarsals and phalanges [16]. There are also intergender differences regarding the alignment of certain osteoarticular segments, as in the angle of the knee [12–14] or in the angle of femoral anteversion [9–11], both with higher values in women.

Ferrari et al. [17] demonstrated in their study on 53 male and 54 female skeletons a greater predisposition of the articular surfaces of the first ray to adduction movements in women, with an orientation of the first metatarsal in adduction.

The gender-dependent differences found by various authors regarding the rotational functionality of the lower extremity, and more specifically, the angle of gait, have been of little clinical significance according to the studies reviewed. Authors such as Murray et al. [20, 21] and Lafuente et al. [19] found a difference of between approximately 1 and 1.5°, obtaining higher values in the group of men. The studies of Seber et al. [22] and Dougan [23] on the angle of gait in men, and that carried out by Patek [24] on the angle of gait in women, independently note intergender differences of the same sense and magnitude.

There are, however, different parameters of the female skeleton that determine a functionality of the lower extremity with internal alignment, such as the greater angle of femoral anteversion [9–11] and the greater internal rotation of the hip [18]. These anthropometric differences, compared and evaluated by physical exploration, do not present a significant clinical impact regarding a reduction in the angle of gait of women with respect to that of men, so there must be other parameters within the female skeleton that determine an external alignment of the extremity. The more transverse metatarsophalangeal joint line of the female forefoot, as shown by the results of this study, could be understood as a determining element in opening the angle of gait in women. As Rueda [37] states, oblique metatarsal parabola may determine internal rotation of the hip as a compensation mechanism. This internal rotation of the hip acts closing the angle of gait. However, another type of study would be necessary to evaluate the relationship between the alignment of the forefoot in the transverse plane and the angle of gait in men and women.

Finally, the reliability of measurements using Autocad (Table 5) shows the propriety of using this software for radiographic measurements.

## 4. Conclusions

There are thus sufficient studies demonstrating anatomical differences in the lower extremity between men and women. The results of the present study should be confirmed with further research in a larger sample, and in which this possible difference of the metatarsal parabola between men and women is correlated with differences in the functionality of the lower extremity.

Different metatarsal parabola between men and women should be considered when designing foot orthoses and shoes. This anatomical difference could be related with lower limb function in male and female biomechanics.

Normal data of metatarsal protrusion contained in this paper are helpful in foot studies in order to set an orthopaedic or surgical treatment of forefoot deformities and pathologies.

## Figures and Tables

**Table 1:** Mean values of metatarsal protrusion related to II ray (in millimeters). Whole specimen.

*N* = 169	II-I	II-III	II-IV	II-V
Mean* ± SD	0.94 ± 2.71	−4.44 ± 1.63	−11.17 ± 2.21	−20.21 ± 3.77
Upper LIM	1.35	−4.20	−10.75	−19.63
Lower LIM	0.53	−4.69	−11.58	−20.78

*IC 95%.

Positive values of metatarsal protrusion mean lengthening of the metatarsal related to II ray expressed in millimeters.

Negative values of metatarsal protrusion mean shortening of the metatarsal related to II ray expressed in millimeters.

**Table 2:** Mean values of metatarsal protrusion related to II ray (in percentage terms). Whole specimen.

*N* = 169	II-I	II-III	II-IV	II-V
Mean* ± SD	0.83 ± 2.19	−3.60 ± 1.22	−9.05 ± 1.89	−16.40 ± 2.52
Upper LIM	1.16	−3.41	−8.76	−16.02
Lower LIM	0.50	−3.78	−9.33	−16.79

*IC 95%.

Positive values of metatarsal protrusion mean lengthening of the metatarsal related to II ray expressed in percentage terms.

Negative values of metatarsal protrusion mean shortening of the metatarsal related to II ray expressed in percentage terms.

**Table 3:** Gender differences in metatarsal protrusion. Mean values of metatarsal protrusion expressed in millimeters related to II ray.

	Female (*N* = 72)	Male (*N* = 97)	Significance
II-I length	1.43 ± 2.17	0.58 ± 3.00	0.034*
II-III length	−3.87 ± 1.27	−4.87 ± 1.73	<0.0005**
II-IV length	−9.60 ± 2.08	−12.33 ± 2.54	<0.0005**
II-V length	−17.88 ± 2.65	−21.93 ± 3.55	<0.0005**

*Significant difference (*P* < 0.05).

**Significant difference (*P* < 0.001).

**Table 4:** Gender differences in metatarsal protrusion. Mean values of metatarsal protrusion expressed in percentage terms related to II ray.

	Female (*N* = 72)	Male (*N* = 97)	Significance
II-I Length	1.27 ± 1.88	0.50 ± 2.35	0.023*
II-III Length	−3.36 ± 1.04	−3.77 ± 1.31	0.029*
II-IV Length	−8.34 ± 1.69	−9.57 ± 1.87	<0.0005**
II-V Length	−15.54 ± 2.07	−17.05 ± 2.64	<0.0005**

*Significant difference (*P* < 0.05).

**Significant difference (*P* < 0.001).

**Table 5:** Intraclass coefficient of correlation values.

*N* = 8	CCI*	Lower LIM	Upper LIM
HV angle (°)	0.986	0.952	0.997
Inter-MTT I-II angle (°)	0.973	0.908	0.994
MTT ADD angle (°)	0.927	0.765	0.984
II RAY length (mm)	1.000	0.999	1.000
II-I length (mm)	0.983	0.945	0.996
II-III length (mm)	0.976	0.922	0.995
II-IV length (mm)	0.977	0.925	0.995
II-V length (mm)	0.996	0.986	0.999

*IC 95%.
